# Echoes of healing: evaluating therapeutic ultrasound in oral submucous fibrosis - An experimental study

**DOI:** 10.6026/973206300210467

**Published:** 2025-03-31

**Authors:** Aparna Raj, Annette M. Bhambal, Palak Choudhary, Rashmi D. Sathe, Karishma Upadhyay, Parnavi Mishra

**Affiliations:** 1 Department of Oral Medicine and Radiology, People's College of Dental Sciences and Research Centre, Bhopal, Madhya Pradesh, India

**Keywords:** Active physiotherapy, jaw-opening exercises, oral submucous fibrosis, therapeutic ultrasound

## Abstract

The effectiveness of therapeutic ultrasound in managing Grade II and III oral submucous fibrosis is of interest. Sixty patients were divided into three groups: ultrasound therapy alone, active physiotherapy alone and a combination of both. Patients were assessed for burning sensation, maximum mouth opening, and cheek flexibility. Statistical analysis (p<0.05) confirmed that combination therapy provided the most significant improvement. The findings suggest that ultrasound therapy combined with active physiotherapy, specifically jaw-opening exercises, is the most effective treatment modality for oral submucous fibrosis.

## Background:

Oral submucous fibrosis is a chronic, debilitating, irreversible condition primarily caused by betel quid chewing [1]. It presents with symptoms such as trismus, burning sensation and difficulty in mouth opening, significantly impacting patients' quality of life [1-2]. Globally, oral submucous fibrosis prevalence is approximately 4.47%, with a higher incidence of 6.36% in India [3]. This is a condition of the oral mucosa that carries a potential for malignancy, with a transformation rate of 4.2% [4]. Alarmingly, over 30% of all cancer cases in India are oral cancers, often linked to oral submucous fibrosis [3]. Management strategies for oral submucous fibrosis include surgical and non-surgical approaches, with the latter encompassing modalities like ultrasound therapy and active physiotherapy ultrasound therapy promotes tissue healing through thermal effects, while active physiotherapy aids in stretching oral muscles. Therefore, it is of interest to evaluate the comparative effectiveness of ultrasound therapy, active physiotherapy and their combination in improving clinical parameters in oral submucous fibrosis patients.

## Methodology:

This study was conducted on 60 oral submucous fibrosis patients visiting the outpatient department of Oral Medicine and Radiology, People's College of Dental Sciences & Research Centre, Bhopal, following institutional ethical approval (IEC No: PCDS/IEC/2024/4/227). Patients aged 20-50 years with clinically confirmed Grade II and III oral submucous fibrosis (as per Nagesh and Bailoor-1993) who had been using pan or other deleterious substances for more than six months and provided informed consent were included. Exclusion criteria encompassed those undergoing oral submucous fibrosis treatment, cases with acute infections or associated lesions (*e.g.*, leukoplakia, carcinoma) and patients with reduced mouth opening due to trauma, surgeries, or established cancer. Participants were randomized using the lottery method into three groups: Group A received ultrasound therapy alone, Group B underwent active physiotherapy with dietary advice to avoid spicy food and Group C received a combination of ultrasound therapy and active physiotherapy Each group followed an 8-week regimen, with ultrasound therapy administered using the Physiotrack machine (1.5 W/cm^2^, continuous mode, 3 MHz, 6 minutes/session). Active physiotherapy included mouth-opening exercises, lateral and protrusive mandibular movements and gradual mouth stretching, performed twice daily for five days a week. Outcomes, including burning sensation, maximum interincisal mouth opening and cheek flexibility (according to Nagesh and Bailoor), were evaluated to determine the therapeutic efficacy of the interventions. Data were analysed using SPSS version 26.0. ANOVA, repeated measures ANOVA and Chi-square tests were applied, with statistical significance set at p<0.05.

## Results:

## Demographic distribution:

Out of 60 participants, 48 were males and 12 females. Grade II oral submucous fibrosis was predominant (44 patients), followed by Grade III (16 patients) Table 1.

At the 2-month mark, inter-group results revealed that Group C (Ultrasound + aPT) exhibited the greatest reduction in burning sensation (1.10 ± 0.85) compared to Group A (1.40 ± 0.50) and Group B (1.20 ± 0.41). Group C (Ultrasound + aPT) showed comparatively better improvement in burning sensation but there was statistically no significant difference found between all three groups (P>0.05). Intra-group results indicated that Group A experienced a significant reduction in burning sensation from baseline (2.20 ± 1.28) to the 2nd month (1.40 ± 0.50) with a p-value of 0.001. Conversely, Group B showed a minimal reduction from baseline (1.30 ± 0.47) to the 2nd month (1.20 ± 0.41), which was not statistically significant (p = 0.163). Group C had the most pronounced reduction from baseline (2.10 ± 1.41) to the 2nd month (1.10 ± 0.85), with a highly significant p-value (0.001) (Table 2).

At the 2-month mark, inter-group results showed that Group C exhibited the highest improvement in cheek flexibility (6.85 ± 2.20), followed by Group A (6.15 ± 2.10), while Group B (5.65 ± 2.27) showed negligible improvement. Group C (Ultrasound + active physiotherapy (APT)) showed comparatively better improvement in Cheek Flexibility, however, there was statistically no significant difference found between all three groups (P>0.05). Intra-group results indicated that Group A significantly improved from baseline (4.55 ± 2.34) to the 2nd month (6.15 ± 2.10) with a p-value of 0.001. Group B had no significant change, with cheek flexibility remaining nearly constant (baseline: 5.65 ± 2.27; 2nd month: 5.65 ± 2.27; p = 0.265). Group C experienced the most substantial improvement from baseline (5.25 ± 2.36) to the 2nd month (6.85 ± 2.20), with a highly significant p-value (0.001) (Table 3).

At the 2-month mark, inter-group results revealed that Group C exhibited the greatest improvement in mouth opening (24.39 ± 4.65 mm), followed by Group A (23.20 ± 5.37 mm), while Group B (22.06 ± 4.29 mm) showed limited improvement. Group C (Ultrasound + active physiotherapy) showed comparatively better improvement in Mouth opening but there was statistically no significant difference found between all three groups (P>0.05). Intra-group results indicated that Group A significantly improved from baseline (21.46 ± 5.61 mm) to the 2nd month (23.20 ± 5.37 mm) with a p-value of 0.001. Group B showed minimal improvement, with mouth opening increasing slightly from baseline (19.62 ± 7.62 mm) to the 2nd month (22.06 ± 4.29 mm), but the change was not statistically significant (p = 0.154). Group C experienced the most substantial improvement, with mouth opening increasing from baseline (22.63 ± 4.47 mm) to the 2nd month (24.39 ± 4.65 mm), with a highly significant p-value (0.001) (Table 4).

## Discussion:

This study assessed the effectiveness of ultrasound therapy in 60 oral submucous fibrosis participants. It was observed that the male to female ratio was 4:1. The reduction in burning sensation, cheek flexibility and mouth opening with ultrasound therapy is likely due to its anti-inflammatory effects, which soften fibrotic tissues with heat and improve collagen fiber extensibility. Ultrasound therapy improves blood flow and removes waste, reducing discomfort from oral submucous fibrosis, while in active physiotherapy; the gentle manipulation of soft tissues increases their flexibility. The findings of this study align with the case report by Vijayakumar and Priya (2013), which showed significant improvement in burning sensation and mouth opening using ultrasound therapy [5]. Similarly, a study by Tyagi *et al.* (2018) demonstrated significant improvements in burning sensation, cheek flexibility and mouth opening with therapeutic ultrasound therapy as an adjuvant [6]. Another study by Dani and Patel (2018) observed greater mouth opening in the experimental group 7]. A study done by Arora and Deshpande the combination of therapeutic ultrasound and jaw opening exercises significantly improved mouth opening and reduced burning sensation in patients with oral submucous fibrosis [8]. The findings of this study also align with the research by Senthilkumar *et al.* which demonstrated a statistically significant improvement in mouth opening among oral submucous fibrosis patients treated with ultrasound therapy and jaw-opening exercises [9]. Shil *et al.* (2024) studied the use of therapeutic ultrasound as an adjunct in oral submucous fibrosis management, the group receiving ultrasound therapy showed the most significant improvement. They concluded that therapeutic ultrasound enhances conventional oral submucous fibrosis treatment [10]. However, a disadvantage of ultrasound therapy is the risk of periosteal burning or pain due to differential heating at tissue interfaces. Fortunately, no cases of such side effects were reported in this study.

## Conclusion:

Therapeutic ultra-sound with active physiotherapy significantly improved burning sensation, cheek flexibility and mouth opening in oral submucous fibrosis patients compared to individual management methods. It is a promising non-surgical treatment modality, warranting further research for broader clinical applications.

## Figures and Tables

**Table 1:** Demographic distribution

**Gender**	**Group A**	**Group B**	**Group C**	**TOTAL**
Male	14	16	18	48
Female	6	4	2	12
**Oral submucous fibrosis Grade**	**Group A**	**Group B**	**Group C**	**TOTAL**
Grade II	12	16	16	44
Grade III	8	4	4	16
TOTAL	20	20	20	60

**Table 2:** Comparative evaluation of burning sensation at baseline to 2nd month (Inter- and Intra-Group Analysis)

**Group**	**Baseline (Mean± SD)**	**1st Week (Mean± SD)**	**1st Month (Mean± SD)**	**5th Week (Mean± SD)**	**2nd Month (Mean± SD)**	**Repeated Measure of ANOVA**
A	2.20± 1.28	2.00± 0.91	1.90± 0.85	1.80± 0.76	1.40± 0.50	9.962	0.001 (HS)
B	1.30± 0.47	1.30± 0.47	1.30± 0.47	1.30± 0.47	1.20± 0.41	2.111	0.163 (NS)
C	2.10± 1.41	1.60± 1.23	1.70± 1.03	1.70± 1.03	1.10± 0.85	8.624	0.001 (HS)
ANOVA 'F' Value	3.79	2.869	2.785	2.242	1.22		
Significance 'P' Value	0.028(S)	0.065(NS)	0.070(NS)	0.116(NS)	0.303(NS)		

**Table 3:** Comparative evaluation of cheek flexibility (baseline to 2 months) (inter- and intra-group analysis)

**Group**	**Baseline (Mean± SD)**	**1st Week (Mean± SD)**	**1st Month (Mean± SD)**	**5th Week (Mean± SD)**	**2nd Month (Mean± SD)**	**Repeated Measure of ANOVA**
A	4.55± 2.34	4.55± 2.34	5.32± 2.10	5.50± 2.15	6.15± 2.10	37.792	0.001 (HS)
B	5.65± 2.27	5.65± 2.27	5.65± 2.27	5.65± 2.27	5.65± 2.27	0.972	0.265 (NS)
C	5.25± 2.36	5.50± 2.36	6.08± 2.30	6.16± 2.30	6.85± 2.20	67.032	0.001 (HS)
ANOVA 'F' Value	1.313	1.313	0.811	0.316	2.307		
Significance 'P' Value	0.277(NS)	0.277(NS)	0.449(NS)	0.730(NS)	0.109(NS)		

**Table 4:** Comparative evaluation of mouth opening (Baseline to 2 Months) (Inter- and Intra-Group Analysis)

**Group**	**Baseline (Mean± SD)**	**1st Week (Mean± SD)**	**1st Month (Mean± SD)**	**5th Week (Mean± SD)**	**2nd Month (Mean± SD)**	**Repeated Measure of ANOVA**
A	21.46± 5.61	21.46± 5.61	22.20± 5.36	22.54± 5.42	23.20± 5.37	158.113
B	19.62± 7.62	21.88± 4.23	21.88± 4.23	21.88± 4.23	22.06± 4.29	2.205
C	22.63± 4.47	22.63± 4.47	23.35± 4.48	23.45± 4.51	24.39± 4.65	187.723
ANOVA 'F' Value	1.259	0.303	0.536	0.55	1.18	
Significance 'P' Value	0.292(NS)	0.740(NS)	0.588(NS)	0.580(NS)	0.315(NS)	
